# Bile Acid Analogs with Anti-Germination Activities for Prophylaxis of *Clostridioides difficile* Infection Alter Bile Acid Homeostasis in the Enterohepatic Cycle

**DOI:** 10.3390/biom15121672

**Published:** 2025-12-01

**Authors:** Nivisa Vakeesan, Efren Heredia, Chandler Hassan, Yang Jiang, Shiv Sharma, Lianyong Su, Huiping Zhou, Steven Firestine, Ernesto Abel-Santos, Wanqing Liu

**Affiliations:** 1 Department of Pharmaceutical Sciences, Eugene Applebaum College of Pharmacy and Health Sciences, Wayne State University, Detroit, MI 48201, USA; gh6788@wayne.edu (N.V.);; 2 Department of Chemistry and Biochemistry, University of Nevada–Las Vegas, Las Vegas, NV 89154, USA; herede1@unlv.nevada.edu (E.H.);; 3 Department of Microbiology and Immunology, School of Medicine, Virginia Commonwealth University and Richmond VA Medical Center, Richmond, VA 23298, USA; lianyong.su@vcuhealth.org (L.S.);; 4 Nevada Institute of Personalized Medicine, University of Nevada–Las Vegas, Las Vegas, NV 89154, USA; 5 Department of Pharmacology, School of Medicine, Wayne State University, Detroit, MI 48201, USA

**Keywords:** *C. difficile*, bile acid, enterohepatic circulation, gut microbiota, transcriptome

## Abstract

We previously reported that two bile acid (BA) analogs, CamSA and CA-Quin, demonstrate potent anti-germination activity against *Clostridioides difficile* (*C. difficile*) spores, protecting rodents from *C. difficile* infections. Here, we further evaluated the impact of these analogs on the hepatic transcriptome and BA homeostasis in vivo by focusing BA profiles on the liver, feces, and chyme as well as the hepatic transcriptome after a 7-day treatment. The two compounds demonstrated similar impact on BA profiles among the three samples, with significantly increased BA excretion in feces. This change is aligned with significantly altered expression of genes involved in BA homeostasis in both liver and gut tissues. Also, both compounds increased levels of deconjugated BAs in the feces, possibly suggesting increased activity of gut microbiota. Fecal levels of anti-*C. difficile* germination chenodeoxycholic acid and pro-germination taurocholic acid are significantly increased and decreased by the treatments, respectively. The hepatic transcriptome showed limited difference in gene expression between the three groups, suggesting a minimal adverse impact of the two compounds on liver function. Overall, our study suggests that in vivo CamSA and CA-Quin treatment demonstrated safe and significantly altered BA homeostasis that inhibits *C. difficle* germination.

## 1. Introduction

*Clostridioides difficile*, (*C. difficile*), is a species of bacteria that is the world’s leading cause of antibiotic-associated diarrhea [1]. *C. difficile* infection (CDI) is mainly spread in health-care settings; however, community spread of CDI is increasing [1,2,3]. About 500,000 people are infected with *C. difficile* every year, with an almost 6% mortality rate [1,2,3].

CDI symptoms range from mild diarrhea to deadly colitis, with the variable symptomatology being attributed to individual susceptibility and complex antibiotic regimes [4]. Primary CDI is mainly treated with vancomycin or fidaxomicin, but up to 20% of patients experience relapses [5].

*C. difficile* is spread by spores, which are dormant and resistant structures that allow *C. difficile* to survive harsh conditions for long periods. *C. difficile* spores germinate in the colon of susceptible patients, a process required to initiate CDI [1,2,3]. Once spores germinate in the antibiotic-altered gut of patients, they generate toxin-producing vegetative cells [4]. Given the risk of antibiotic resistance and treatment-induced dysbiosis of the gut microbiota, there is an urgent need to discover new therapeutics to treat CDI.

The germination of *C. difficile* spores is mediated by several factors including the composition of bile acids in the gut. Taurocholate (TCA), a conjugated primary bile acid, facilitates germination while chenodeoxycholate (CDCA), an unconjugated primary bile acid, inhibits it [6,7]. Bile acids are synthesized in the liver and secreted into the intestines, where their primary function is to emulsify and facilitate the absorption of fats and vitamins [8,9]. Bile acids synthesized endogenously in the liver are classified as primary bile acids. In humans, these are cholic acid (CA) and chenodeoxycholic acid (CDCA) [8,9]. Mice have three additional endogenous bile acids: ursodeoxycholic acid (UDCA), alpha-muricholic acid (αMCA), and beta-muricholic acid (βMCA) [8]. Once primary bile acids are synthesized in hepatocytes, either taurine (T) or glycine (G) are attached to the carboxylic acid to form conjugated bile acids which require specific transporters to move across the canalicular membranes [8,9]. Once bile acids reach the intestinal ileal lumen, bacteria can deconjugate and dehydroxylate them into secondary bile acids lithocholic acid (LCA) and deoxycholic acid (DCA), respectively [8,9]. By the time they reach the end of the ileum, about 95% of the bile acids are reabsorbed by enterocytes and transported back to the liver through enterohepatic circulation (EHC), which continuously recycles bile acids between the intestines and the liver [8,9]. Secondary bile acids are absorbed from the intestine and reconjugated in the liver before re-entering the EHC [9].

Since bile acids-mediated spore germination is required for CDI, we expect that anti-germination therapy will prevent CDI and its relapse [10]. We have tested synthetic bile salt (SBS) libraries and found analogs that inhibit *C. difficile* spore germination [11,12,13,14]. More importantly, SBS anti-germinants also prevented CDI in rodents [15,16,17,18] without any overt toxicity seen after a 30-day treatment. More specifically, we found that cholic acid substituted with *m*-aminosulfonic acid (CamSA) [19,20] and quinazoline cholate analog (CA-Quin), inhibit *C. difficile* spore germination at micromolar concentrations even in the presence of saturating millimolar levels of the germinant taurocholate. Given that CamSA was the first CDI prophylactic discovered in our group, it has been extensively characterized both in vitro and in vivo [14,15,16,17,18,21,22].

One surprising finding was that a single dose of CamSA was sufficient to protect mice from CDI. To study this intriguing pattern, we examined the pharmacokinetics of CamSA [21]. Our data suggests that CamSA undergoes EHC. We hypothesize that the cycling of CamSA between the liver and intestines serves as a slow-release mechanism that allows CamSA to be retained in the gastrointestinal tract for days. This model explains how a single CamSA dose can provide multi-day protection against murine CDI [18]. Although CA-Quin also protects mice from CDI, we have not determined whether it enters the EHC.

Given the involvement of CamSA in the EHC and the fact that these agents are bile salt analogs, it is unclear whether they affect regular circulation or metabolism of natural bile acids. It also remains to be further assessed whether CamSA and CA-Quin have toxicological impacts on the liver. In this study, we treated mice with CamSA, CA-Quin, or vehicle control for 7 days. The liver transcriptome and bile acid profiles of the liver, ileum, chyme, and feces samples were analyzed and compared. In conjunction with previous studies, the present study indicates that the two bile acid analogs are safe and alter bile acid profiles in a manner benefiting *C. difficile* inhibition. These findings warrant further investigation into their potential for the clinical treatment of CDI.

## 2. Materials and Methods

### 2.1. Materials

All materials used in the assays of this study are summarized in Supplementary Table S1.

### 2.2. Synthesis of CamSA and CA-Quin

The synthesis of CamSA [19] and CA-Quin [11] were performed according to previously published methods by the Dr. Firestine lab at Wayne State University, Detroit, MI, USA.

### 2.3. Animals

Animal protocols were performed in accordance with the Guide for Care and Use of Laboratory Animals outlined by the National Institutes of Health. Protocols were reviewed and approved by the Institutional Animal Care and Use Committee (IACUC) at the University of Nevada, Las Vegas (Permit Number: R0914-297, approval date: 20 June 2025). Weaned male C57BL/6J mice were obtained from Jackson Labs, Jax West (Bar Harbor, ME, USA). Mice were housed in groups of five mice per cage at the University of Nevada, Las Vegas, animal care facility. Upon arrival at the facility, mice were quarantined and allowed to acclimate for at least one week prior to experimentation. Animals were 5–8 weeks old and were between 20 and 25 g. Tissue samples were collected after euthanasia. Therefore, no anesthesia was utilized. Drugs were administered by oral gavage and dissections were conducted after euthanasia.

### 2.4. Anti-Germinant Treatment and Organ Harvest

Male mice (*n* = 4 per group) were treated with a once-daily dose of DMSO, 50 mg/kg CamSA or 50 mg/kg CA-Quin for 7 consecutive days. Feces were collected from individual animals after 3 days and 7 days of treatment. On day 7, all animals were euthanized with CO_2_, and organs were harvested as follows.

Livers from each animal were harvested and weighed. A liver slice (not wider than 0.5 cm) was stored in cold RNAprotect Tissue Reagent (Qiagen, Venlo, The Netherlands). A second part (200–300 mg) was chopped, placed into aluminum foil, and flash frozen. Foils were then transferred into pre-cooled Eppendorf tubes and stored at −80 °C.

A 15 cm segment of the ileum proximal to the ileocecal valve was cut from each animal and flushed with extraction buffer (0.1 M Tris-buffered saline with 0.3% bovine serum albumin, 0.01% sodium azide, and 0.002% Tween). The resulting intestinal content suspensions (chyme) were flash frozen and stored at −80 °C. The cleaned ileums were sliced lengthwise and cut into equal sized pieces. One piece was stored in cold RNAprotect Tissue Reagent (Qiagen, Venlo, The Netherlands). Three other ileum pieces were put into foil, as above.

### 2.5. RNA Extraction

Frozen liver samples stored in Qiagen RNAprotect Tissue Reagent (Qiagen, Venlo, The Netherlands) were homogenized, and RNA was extracted utilizing the Qiagen RNeasy Kit (Qiagen, Venlo, The Netherlands) by following the manufacturer’s protocol. Additional flash frozen liver and ileum samples were homogenized in TRIzol reagent (Invitrogen by Thermo-Fisher Scientific, Santa Clara, CA, USA) and extracted by following manufacturer’s protocol.

### 2.6. Sequencing

Briefly, total RNA was extracted from frozen liver and ileum samples using TRIzol reagent (Invitrogen by Thermo-Fisher Scientific, Frederick, MD, USA) followed by purification steps as detailed by the manufacturer. RNA purity and quantity were assessed via spectrophotometer (NanoDrop 8000, Thermo-Fisher Scientific, Frederick, MD, USA). Single end RNA sequencing was performed by the Wayne State University Genome Sciences Core, Detroit, MI, USA. The mRNA-seq library was prepared using the QuantSeq 3′ mRNA-Seq Library Prep Kit FWD (Lexogen, Greenland, NH, USA). Libraries were assessed by the High Sensitivity D1000 (HS D1000) ScreenTape Assay (Agilent, Santa Clara, CA, USA). Samples were sequenced on the NovaSeq system (Illumina, San Diego, CA, USA).

### 2.7. RT-PCR and qPCR

Extracted RNA from frozen tissue was utilized for RT-PCR which was performed utilizing Thermo-Fisher High-Capacity cDNA Revere-Transcription kit (Applied Biosystems by Thermo-Fisher Scientific, Frederick, MD, USA). qPCR was conducted utilizing the Thermo-Fisher PowerUp SYBR Green Master Mix for qPCR (Applied Biosystems by Thermo-Fisher Scientific, Frederick, MD, USA) for the liver samples and ileum samples (in technical triplicates) in a QuantStudio qPCR machine (Applied Biosystems by Thermo-Fisher Scientific, Frederick, MD, USA) utilizing manufacturer’s protocol. Primer sequences are included in Supplementary Table S2.

### 2.8. LC-MS of Bile Species

LC-MS was performed by the Zhou lab at the Department of Microbiology and Immunology, School of Medicine, Virginia Commonwealth University and Richmond VA Medical Center, Richmond, VA, USA. All bile acids were extracted and quantified by LC-MS/MS as previously described [23,24]. The following bile acids were measured (in nmol/g): 12-keto-lithocholic acid (12-keto-LCA), 3-keto-7α,12α-dihydroxy-5β-cholan-24-oic acid (3-keto,7α,12α(OH)_2_), 3-keto-lithocholic acid (3-keto-LCA), 7-keto-deoxycholic acid (7-keto-DCA), 7-keto-lithocholic acid (7-keto-LCA), allo-isolithocholic acid (allo-isoLCA), alpha-muricholic acid (α-MCA), beta-muricholic acid (β-MCA), 7α-hydroxy-4-cholesten-3-one (C4), cholic acid (CA), cholic acid-3-sulfate (CA-3-S), cholic acid-7-sulfate (CA-7-S), chenodeoxycholic acid (CDCA), chenodeoxycholic acid-3-sulfate (CDCA-3-S), deoxycholic acid (DCA), deoxycholic acid-3-sulfate (DCA-3-S), glyco-beta-muricholic acid (Gβ-MCA), glycocholic acid (GCA), glycochenodeoxycholic acid (GCDCA), glycodeoxycholic acid (GDCA), glycohyocholic acid (GHCA), glycohyodeoxycholic acid (GHDCA), glycolithocholic acid (GLCA), glycoursodeoxycholic acid (GUDCA), hyocholic acid (HCA), hyodeoxycholic acid (HDCA), isodeoxycholic acid (isoDCA), isolithocholic acid (isoLCA), lithocholic acid (LCA), lithocholic acid-3-sulfate (LCA-3-S), murideoxycholic acid (MDCA), tauro-alpha-muricholic acid (Tα-MCA), tauro-beta-muricholic acid (Tβ-MCA), taurocholic acid (TCA), taurochenodeoxycholic acid (TCDCA), taurodeoxycholic acid (TDCA), taurohyodeoxycholic acid (THDCA), taurolithocholic acid (TLCA), tauroursodeoxycholic acid (TUDCA), tauro-omega-muricholic acid (Tω-MCA), ursodeoxycholic acid (UDCA), ursodeoxycholic acid-3-sulfate (UDCA-3-S), and omega-muricholic acid (ω-MCA). Samples were filtered (0.2 µm PTFE) and analyzed using a Shimadzu (Columbia, MD, USA) LCMS-8600 system.

### 2.9. Differential Gene Expression Analysis

Sequencing data from the Wayne State University Genomics Core, Detroit, MI, USA was analyzed for quality by FastQC [25] and then trimmed by Trimmomatic [26] and rechecked for quality. RNA reads were mapped to the mouse genome utilizing Hisat2 [27]. Gene counts were collected utilizing HTSeq [28,29] and then the data was analyzed in R (version 4.4.2) [30] utilizing RStudio (2024.9.1.394) [31]. In R, DESeq2 [32] was utilized to perform differential gene expression and obtain normalized counts. Ggplot2 [33] was utilized to generate the principal component analysis and volcano plots. Venn diagram plotted using ggvenn [34]. Complex heatmap [35,36] was utilized for the heatmaps. KEGGREST [37], biomaRt [38,39], AnnotationDbi [38], and org.Mm.eg.db [39] were utilized for gene annotation and pathway analysis. Ggrepel [40] and ggprism [41] were utilized in improving and customizing visualization of various figures. Openxlsx [42] was utilized to export data to Excel.

### 2.10. Enrichment Analysis

For enrichment analysis, significant differentially expressed genes (DEG) with a log2 fold change +/− 1.5 and adjusted *p* < 0.05 for the respective treatment group were used. Enrichment analysis was conducted utilizing Metascape (v3.5.20250701) [43], a custom gene ontology (GO) analysis. We also performed a custom TRRUST [44] enrichment analysis in Metascape [43] to investigate the potential key upstream regulators for the significant DEGs (unadjusted *p* < 0.05). The significance cut-offs for enrichment analysis were a *p* value of less than 0.01, minimal overlap with three genes, and minimum enrichment of 1.5.

### 2.11. Statistical Analysis

Concentrations provided by LC-MS-based measurement were utilized. Bile acids concentration (nmol/g) data were log2 transformed for normality. For bile acid ratio, data were log2 transformed before calculating the ratio. Percent composition was calculated by diving the concentration of individual species by the total concentration quantified in a sample. For qPCR assays, 2^−ΔΔCT^ was quantified utilizing Microsoft Excel (Version 16.94 (25020927), 2025 Microsoft) before transferring data for statistical analysis. No corrections were applied to *p* values for *t* tests. Ordinary one-way ANOVA, followed by the post hoc Tukey tests (that have considered appropriate adjustment for multiple testing), and data visualization were conducted utilizing GraphPad Prism (Version 10.4.1(532), GraphPad Software, LLC, Solana Beach, CA, USA). No corrections were applied to *p* values for *t* tests. (**** *p* < 0.0001, *** *p* < 0.001, ** *p* < 0.01, * *p* < 0.05)

Ggplot2 [33] and ggprism [41] were utilized to generate the principal component analysis. Vegan [45] and boot [46,47] were utilized for PERMANOVA analysis. Dplyr [45] and openxlsx [42] were utilized to prepare and export data to Excel. Detailed statistics including mean, standard deviation, and statistical significance of bile salt level comparison are available upon request.

## 3. Results

To investigate the effects of the bile acid analogs on the EHC, mice were treated with either DMSO, CamSA, or CA-Quin for seven consecutive days. Bile acid species (*n* = 43) of the liver (*n* = 4) and chyme (*n* = 4) were profiled on day 7 utilizing LC-MS/MS. Additionally, feces from days 3 (*n* = 4) and 7 (CamSA *n* = 3, CA-Quin *n* = 4, and DMSO *n* = 4) of treatment were also analyzed. A total of 42 bile acids were quantified; of those, there were 5 primary bile acids, 9 conjugated primary bile acids, 20 secondary bile acids, and 8 conjugated secondary bile acids. The categorization of the bile acids detected by LC-MS is indicated in Supplementary Table S3.

In addition to the 42 bile acids, 1 bile acid precursor was also quantified, 7-α-hydroxy-4-cholesten-3-one (C4). The bile acids were divided according to where or how they are synthesized. Primary bile acids are produced in the liver and then conjugated with either taurine or glycine to form the conjugated primary bile acids. The secondary bile acids are derived from primary bile acids by bacterial biotransformation. For analysis of the bile acids, only bile salts that were quantified in at least three samples of one treatment group were included for analysis. In Supplementary Table S4, we have indicated how many bile acids were at quantifiable levels for each treatment.

### 3.1. Overall Alterations of Bile Acids by CamSA and CA-Quin in the Liver, Chyme, and Fecal Samples

**Figure 1:**
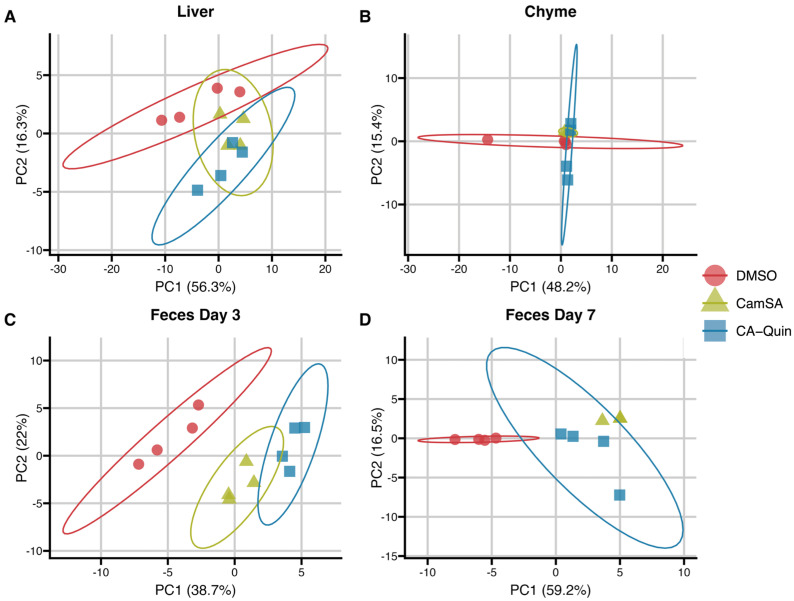
PCA plots of the quantified bile species within each sample; (**A**) liver, (**B**) chyme, (**C**) feces day 3, and (**D**) feces day 7. The raw bile acid concentration was utilized to generate these PCA plots. PERMANOVA/adonis results presented in Supplementary Table S5.

A principal component analysis (PCA) plot of the BA profile data shows significant (*p* value < 0.05) separation between the two treatment groups and the DMSO control group in the feces, but not in the liver, while only the CA-Quin-treated group was significantly separated from the other two groups in the chyme. However, generally large confidence intervals indicate less confidence, potentially due to smaller sample size (Figure 1, Table S5).

**Figure 2:**
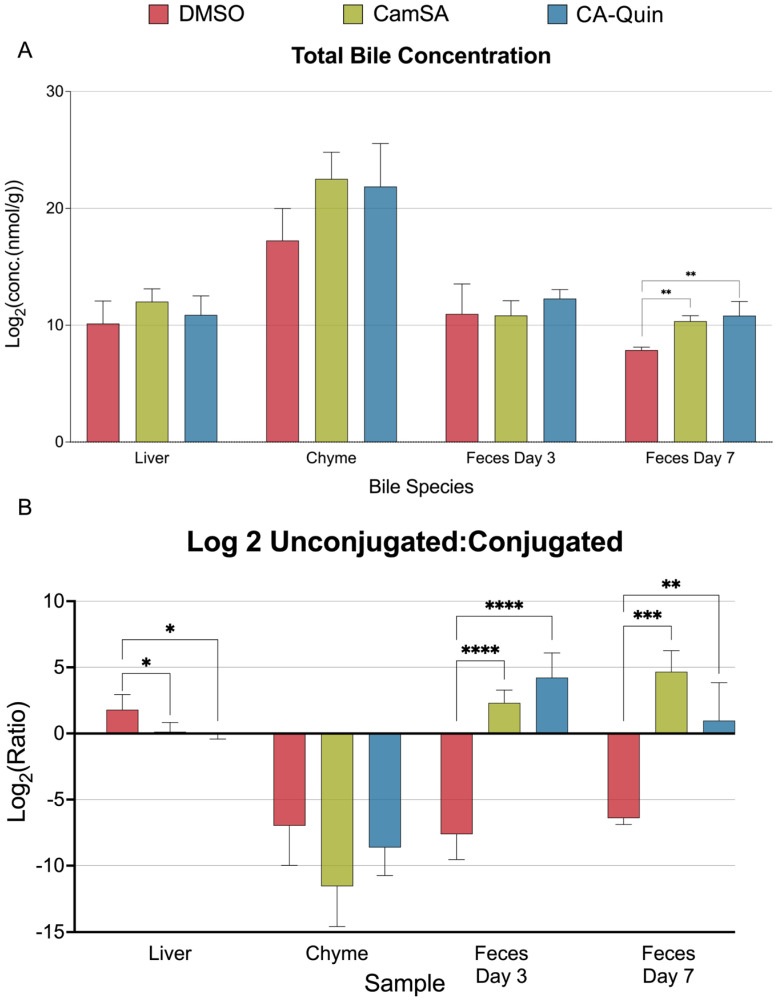
Overall statistics of bile acid quantifications for the four tissue samples. (**A**) The total concentration (nmol/g) of bile acids quantified in each sample was summed up and log2 transformed. (**B**) Ratio between the total level of unconjugated bile acids and total level of conjugated bile acids. Statistics were based on the post hoc Tukey pair-wise test under ordinary one-way ANOVA after the ratio was log2 transformed. (**** *p* < 0.0001, *** *p* < 0.001, ** *p* < 0.01, * *p* < 0.05).

Neither CamSA nor CA-Quin altered the total level of bile acids compared to the DMSO-treated group among the liver, chyme, and feces collected on day 3. However, total BA content significantly increases in the feces collected on day 7 of both treatment groups (Figure 2A). When comparing the ratio of total unconjugated and conjugated BAs, both treatment groups demonstrated a significant trend towards lower ratios of unconjugated–conjugated BAs in the liver whereas higher ratios of unconjugated–conjugated BAs were noted in the feces, compared to the control group (Figure 2B).

**Figure 3:**
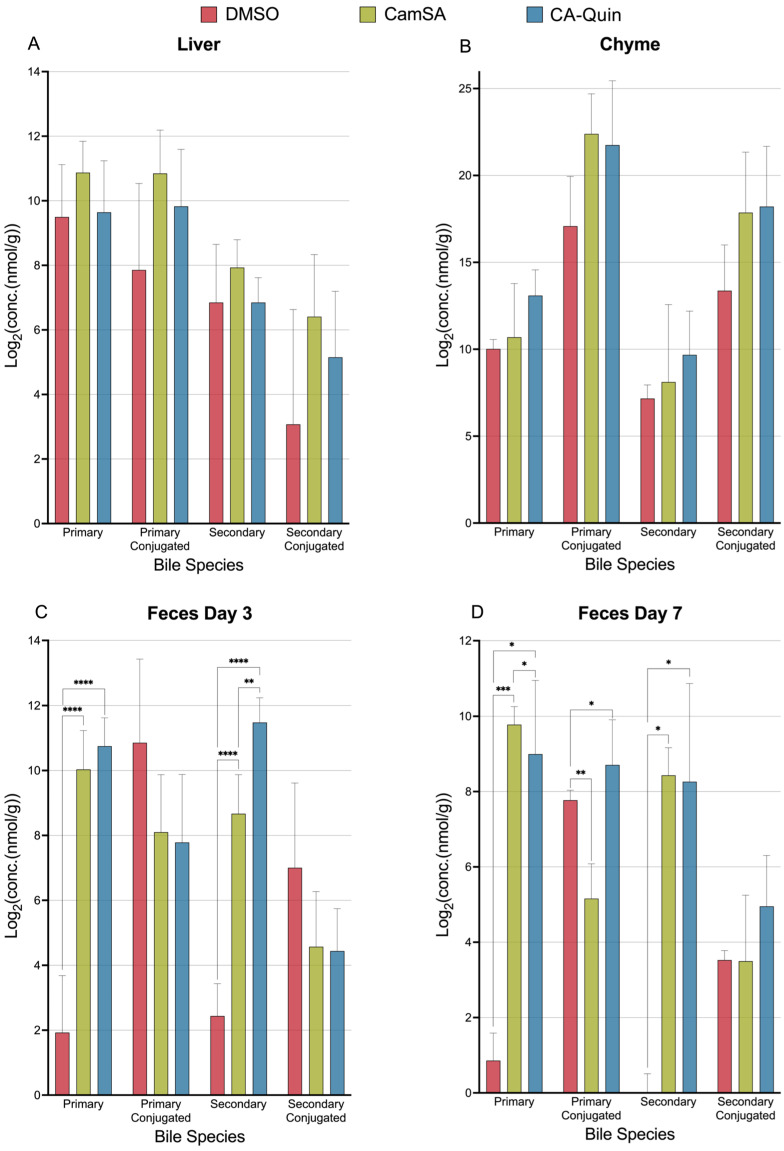
Concentration (nmol/g) levels of the total bile acids in four categories for each tissue sample. (**A**) liver; (**B**) chyme; (**C**) feces Day 3; and (**D**) feces Day 7. Statistics were based on the post hoc Tukey pair-wise test under ordinary one-way ANOVA after the ratio was log2 transformed. (**** *p* < 0.0001, *** *p* < 0.001, ** *p* < 0.01, * *p* < 0.05).

When analyzing the changes in BA categories (primary, primary conjugated, secondary, and secondary conjugated), CamSA or CA-Quin treatments did not significantly change the total BA levels of each category in the liver or chyme (Figure 3A,B). In contrast, both unconjugated primary and secondary BAs are significantly increased in the feces (both day 3 and day 7) of both treatment groups compared to the control group (Figure 3C,D). In the feces collected on day 7, the conjugated primary BAs are significantly underrepresented in the CamSA-treated group and overrepresented in the CA-Quin-treated group (Figure 3D).

#### Alternation of Individual BA Levels y CamSA and CA-Quin Treatment

**Figure 4:**
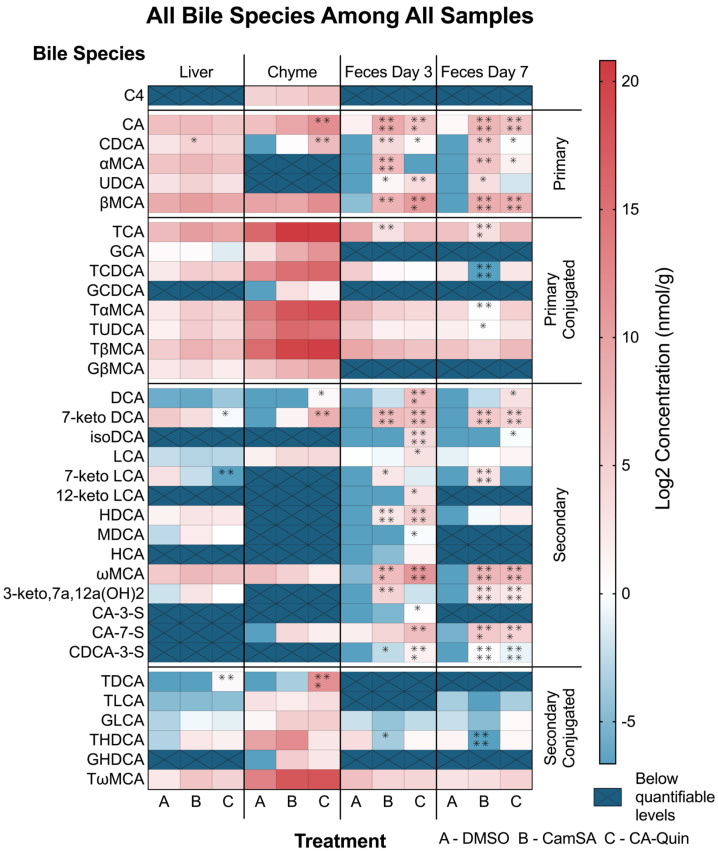
Level of each bile acid within the four tissue samples. Statistics were based on the post hoc Tukey pair-wise test under ordinary one-way ANOVA after the ratio was log2 transformed (**** *p* < 0.0001, *** *p* < 0.001, ** *p* < 0.01, * *p* < 0.05). Heatmap was generated based on the log2 transformed data. Hashed cells denote bile acid species undetectable in the sample.

Analysis of individual bile acid concentration changes showed that the major significant changes in the level of BAs are in the fecal samples of animals treated with the two compounds, with fewer significant changes observed in the liver or chyme samples (Figure 4). Nearly all five primary BAs demonstrated an increased level in the feces (both day 3 and day 7) of both CamSA- and CA-Quin-treated mice, compared to the DMSO-treated control group. In contrast, only CDCA increased in the liver of CamSA-treated mice, while both CA and CDCA increased in the chyme of CA-Quin-treated mice (Figure 4).

No significant change was observed for conjugated BAs in the liver tissue for any of the treatment groups compared with the DMSO group. In the feces samples, all conjugated BAs showed some decrease in both CamSA- and CA-Quin-treated mice on day 3, but only TCA showed a statistical significance in the CamSA group. On day 7, feces showed that more species (TCA, TCDCA, TaMCA, and TUDCA) had a statistically significant decrease in the CamSA-treated (but not the CA-Quin-treated group) mice (Figure 4). As TCA and CDCA have been demonstrated to be directly involved in *C. difficile* activation, we compared the TCA:CDCA ratio between the three groups among the four samples. A significant decrease in this ratio was noted in both treatment groups on day 3. On day 7, only the CamSA-treated mice showed a significant decrease in this ratio (Figure S1).

Among the 14 secondary BAs that were examined, 7-keto-DCA and 7-keto-LCA levels showed a significant decrease in the liver tissue of mice treated with CA-Quin. The same group also demonstrated a significantly increased level of DCA and 7-keto-DCA in the chyme. In contrast, most of the detectable secondary BAs in feces showed a statistical increase on day 3 and day 7 in both the CamSA- and CA-Quin-treated groups (Figure 4).

Only a few conjugated secondary BAs demonstrated statistically significant changes. TDCA levels in the liver and chyme of the CA-Quin group are significantly increased, while THDCA levels significantly decreased in day 3 fecal samples of the CamSA group and in day 7 fecal samples of the CA-Quin group.

Besides the absolute concentration as demonstrated above, we also further examine the relative composition of individual BAs among the samples (Figure S2). While the pattern of the relative level of BAs largely resembles that of the absolute BA concentration shown in Figure 4, several BAs demonstrated stronger association with the two treatments. For example, compared to the control group, the relative TCA level in both treatment groups is significantly increased and decreased in both the liver and fecal samples, respectively (Figure S2).

### 3.2. Liver Transcriptomics

**Figure 5:**
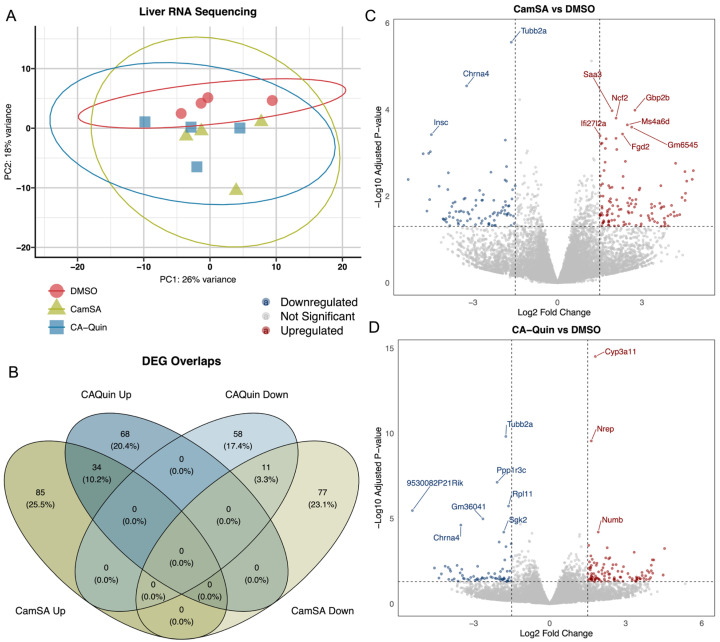
Hepatic transcriptomic analyses. (**A**) PCA plot of the overall hepatic transcriptomic data of the three groups. (**B**) Venn diagram showing the number of significantly (log2 fold change +/− 1.5 with adjusted *p* < 0.05) up- and downregulated genes as well as shared genes in each group. “Down” indicates the downregulated genes and “Up” indicates the upregulated genes. (**C**,**D**) Volcano plots of the significant DEGs for CamSA and CA-Quin treatment groups, respectively. Highlighted genes are those with a log2 fold change +/− 1.5 and adjusted *p* < 0.05. Data are presented as −log10 (adjusted *p* value). The highlighted genes in blue were downregulated while the genes in red were upregulated as compared to the control group.

To examine the impact of CamSA and CA-Quin treatments on the murine liver transcriptome, RNA sequencing was performed [DMSO (*n* = 4), CamSA (*n* = 4), CA-Quin (*n* = 4)]. PCA plots showed overall minimal significant changes in the hepatic transcriptome program due to CamSA or CA-Quin treatments (Figure 5A, Table S5).

#### Genes and Transcriptome Patterns Associated with Drug Treatments

All significant DEGs were included in Table S6. A total of 119 and 102 genes are significantly (|log2 fold change| > 1.5 and adjusted *p* < 0.05) upregulated in the CamSA- and CA-Quin-treated groups compared to the control, respectively. Among these, 34 are shared between the two treatment groups. Similarly, a total of 88 and 69 genes, respectfully, are significantly downregulated in the CamSA- and CA-Quin-treated groups compared to the control. Among these genes, 11 were shared between the two treatment groups. No genes that are downregulated in the CamSA group were upregulated in the CA-Quin treatment, while no genes upregulated in the CamSA group were downregulated in the CA-Quin treatment (Figure 5B–D).

To further explore insights into the potential pharmacological effects related to the two treatments, we performed pathway enrichment analyses by focusing on these significant differentially expressed genes (DEGs). The Metascape [43] enrichment analysis (Figure S3) showed that the top gene ontology (GO) pathways enriched in the CamSA-related upregulated DEGs include *regulation of attachment of spindle microtubules to kinetochore, chemotaxis, and taxis.* The only CamSA-related downregulated DEG is *spermatogenesis.* The top GO pathways enriched in CA-Quin related DEGs that were upregulated include *lateral ventricle development, cellular response to interferon-alpha, and inner ear receptor cell development.* The CA-Quin related downregulated pathways include *modulation of excitatory postsynaptic potential, regulation of stem cell population maintenance, and placenta development.* Shared upregulated GO pathways include *defense response to virus, response to virus, and regulation of innate immune response.* There were no shared downregulated GO pathways (Figure S3C).

### 3.3. Impact of Drug Treatment on Key Pathways in the Liver

**Figure 6:**
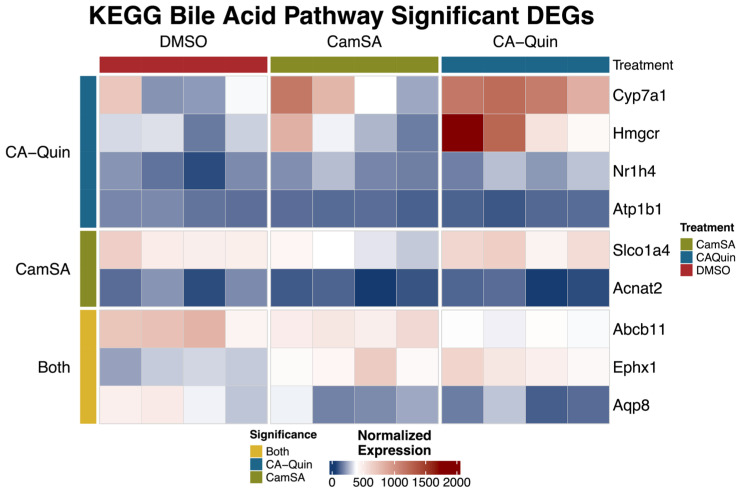
Expression pattern of genes involved in bile synthesis and secretion pathways. Shown here are normalized expression (normalized gene count) levels of significant (unadjusted *p* < 0.05) DEGs between at least one treatment group and the control group. Statistical significance (unadjusted *p* < 0.05) in each treatment was indicated by the color bar on the left.

Both CamSA and CA-Quin are CA analogs and CamSA have been demonstrated to enter the EHC. Meanwhile, both treatments led to significantly altered BA profiles as noted above. Therefore, we conducted a targeted analysis on the expression of key genes involved in BA homeostasis based on the gene list of the “*bile acid synthesis and bile secretion*” pathway in KEGG [48,49,50]. We examined the DEGs by relaxing the significance level of DEGs (unadjusted *p* < 0.05) given the modest gene expression changes. Notably, genes encoding key enzymes involved in cholesterol (*Hmgcr*) and BA biosynthesis [*Cyp7a1* and *Fxr* (*Nr1h4*)] are significantly upregulated in the CA-Quin-treated group compared to the control group, while *Bsep* (*Abcb11*), the bile acid efflux transporter gene, is significantly downregulated among both drug treatment groups (Figure 6).

To further understand the potential mediators for the drug treatment impact on the liver, we also performed an enrichment analysis to identify potential transcription factors regulating the significant DEGs (unadjusted *p* < 0.05). We found that *constitutive androstane receptor* (*Car*/*Nr1i3*) and *peroxisome proliferator-activated receptor alpha* (*Pparα*) are the two transcription factors significantly enriched for potentially regulating upregulated DEGs in the CA-Quin-treated group but not in the CamSA-treated group (Figure S4A). The *Car-* and *Pparα*-target genes including *Cpt1a*, *Cyp3a11*, *Cyp7a1*, *Cebpb*, *Pdk4*, and *Retsat* (Figure S4B).

### 3.4. qPCR Quantification of BA Metabolism and Transporter Genes in the Liver and Ileum

**Figure 7:**
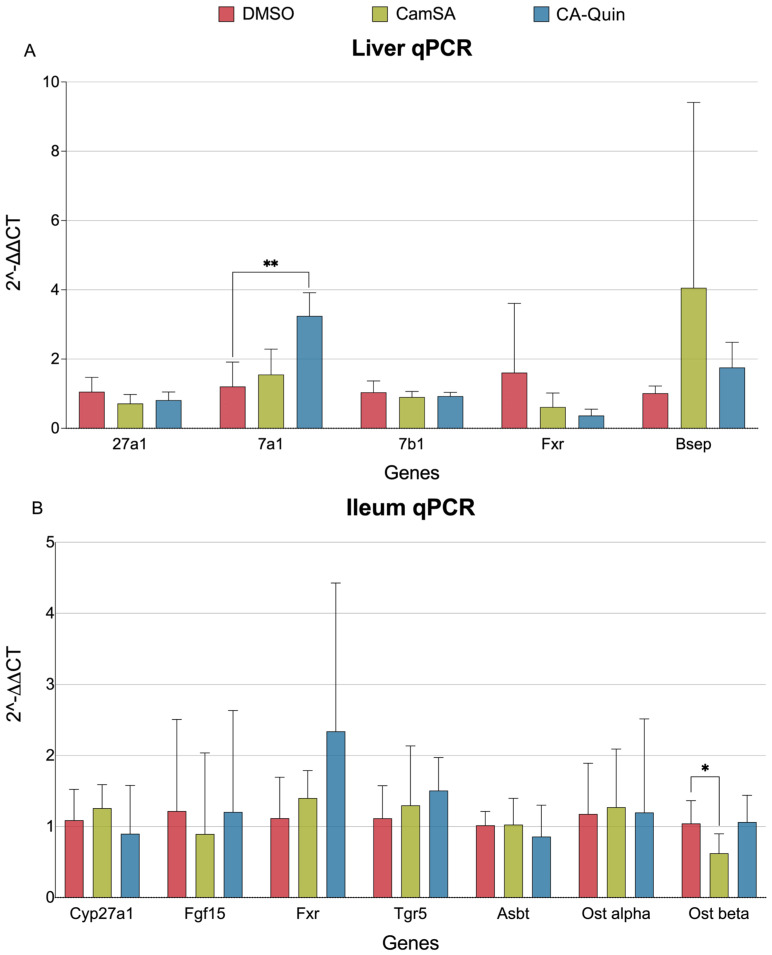
Relative expression level quantified with qPCR of genes involved in bile synthesis, transport, and signaling. (**A**) Respective 2^−ΔΔCT^ results for genes within liver. (**B**) Respective 2^−ΔΔCT^ results for genes within ileum. Statistics were based on the post hoc Tukey pair-wise test under ordinary one-way ANOVA. (** *p* < 0.01, * *p* < 0.05).

To validate the transcriptome results, qPCR was performed for selected genes involved in bile synthesis and transport. *Cyp7a1* expression was significantly increased in CA-Quin-treated animals, while no other gene was significant. Similarly to the transcriptomic data, qPCR showed that expression of *Cyp27a1*, *Cyp7b1*, and *Fxr* (*Nr1h4*) was not significantly altered by either CamSA or CA-Quin (Figure 7A).

EHC transport of bile involves numerous proteins in the ileum. Our qPCR data shows that *Ostβ*, a key transporter involved in bile resorption [51], was significantly downregulated in the ileum from the CamSA-treated mice (Figure 7B).

## 4. Discussion

Previously, we have shown that our first-generation germination inhibitor, CamSA, can be maintained at therapeutic levels due to recycling via the EHC [14,15,21]. However, CamSA can be hydrolyzed by gut bacteria and become inactive and can potentially release toxic byproducts [21]. In addition, one of our other parent inhibitors, CA-Quin, has shown significant potency. While both compounds do not show obvious signs of toxicity, detailed studies on hepatotoxicity and the effects on bile acids have not been performed. To address this issue, we conducted a transcriptomic and bile acid metabolomic analysis on mice treated with the vehicle (DMSO) and the inhibitors, CamSA and CA-Quin, for 7 days.

The minimal alteration of hepatic transcriptome by both treatments as demonstrated in the PCA plots indicates that the potential adverse impact of these two compounds on liver function is very limited. We also found that a considerable proportion of the DEGs associated with each treatment are shared between the two drug-treated groups, indicating that the two compounds may share, at least in part, profiles in pharmacokinetics and pharmacodynamics. On the other hand, there are also some differences in the transcriptomic profiles related to the two compounds. For example, *Car* and *Pparα* are enriched as significant upstream regulators for the upregulated DEGs in the CA-Quin-treated group but not in the CamSA-treated group. Whether CA-Quin directly targets these transcription factors remains to be investigated. We also noticed that upregulated DEGs in the CamSA group are enriched to inflammatory response-related pathways, which are different from that associated with CA-Quin treatment. Whether this leads to potential liver injuries should be further studied. Unfortunately, we failed to obtain high-quality histological data using the available frozen liver, which does not allow us to examine the impact of drug treatments on liver pathology. However, our previous published works have shown that mice and hamsters treated with multi-day high doses of CamSA do not develop any overt signs of acute or sub-chronic toxicity. Furthermore, CamSA-treated mice show no hepatic or intestinal anomalies. Similar lack of toxicity was observed with other bile salt analogs. Similarly, CA-Quin-treated animals in this study did not show overt signs of toxicity or organ alterations [15,16,17,18].

While both compounds are BA analogs, our previous study has demonstrated that CamSA enters the EHC [21]. It is thus logically hypothesized that both compounds may interfere with the EHC and homeostasis of BAs. Examination of samples from mice treated with these bile salt analogs demonstrated a few interesting patterns. First, the overall alteration of all BAs among different tissues are highly similar between the CamSA and CA-Quin treatment compared to the DMSO control, which further suggests a similar impact of the two compounds on BA homeostasis. We also found that the major changes in BA profiles associated with both treatments are the increased BA levels among fecal samples, largely attributed to deconjugated primary and secondary species, with a high similarity between the samples collected at the two timepoints, suggesting an increased excretion of BAs following both treatments. This trend may suggest a decreased re-absorption of BAs from the intestine back to the liver. This is further supported by the reduced levels of a few secondary BA species in the liver and decreased transcription of the transporter *Ostβ* in the CamSA-treated ileum samples.

Reduced BA re-absorption by the EHC may signal to the liver to increase production of BAs. Because BAs are sterol derivatives, the net effect would be an elevated need for cholesterol usage. This is evidenced by the significant upregulation of hepatic expression of *Cyp7a1* (the rate-limiting enzyme controlling the conversion of cholesterol to BA) and *Hmgcr* (rate-limiting enzyme for cholesterol biosynthesis). Furthermore, the downregulation of *Bsep* in both drug-treated groups perhaps also reflects the tendency of BA retention in the liver. However, the increased expression of *Cyp7a1* does not align with the unchanged level of *Fgf15* in the ileum based on the qPCR data. It is also unclear whether increased *Cyp7a1* while decreased *Bsep* would lead to potential cholestatic stress in the liver, especially when there is a long-term treatment. These potential issues should be further clarified in future studies. Nevertheless, the fecal data indicates that the two compounds, especially CA-Quin, likely inhibit BA reabsorption and promote intestinal BA excretion.

More detailed analysis of the BA profiles further demonstrated that intestinal TCA and CDCA levels were significantly altered by the two treatments. TCA and CDCA were demonstrated to contribute to the activation and inhibition of *C. difficile* germination, respectively [19,52]. In this study, we found that the TCA level was significantly reduced in both the chyme and feces samples of the CamSA-treated mice, while the CDCA level was significantly increased in the chyme of CA-Quin group and in feces samples of both treatment groups. The TCA:CDCA ratio is significantly lower among both drug-treated groups compared to the control group in the fecal samples collected on day 3. Therefore, our data accounts for, at least in part, the inhibitory effects of the two compounds in treating CDI.

In a previous pharmacokinetic analysis of CamSA, we found that it is hydrolyzed into CA and metanilic acid (mSA) [21]. Thus, the increase in CA level observed in the CamSA-treated mice may be due to the hydrolysis of the drug. Even though there is an increased level of CA in the ileum of CA-Quin-treated mice as well as in feces samples of both treatments, this increased CA is more likely attributed to the reduced reabsorption. Nevertheless, increased CA in the gut may also be beneficial in treating CDI, as it has been found that CA can bind to one of the *C. difficile* toxins, TcdB, and reduce its toxic effects [53].

Despite no direct observation, our data also indicates an altered gut microbiome following the two treatments. We previously reported that 10 days of treatment with CamSA in uninfected animals resulted in significantly different microbiome diversity [15]. The BA profile changes shown here are consistent with those prior microbiota observations. Because both the de-conjugation of primary BAs and the production of secondary BAs rely on bacterial enzymes [8,53,54,55], the increased level of de-conjugated primary and unconjugated secondary BAs in the feces of treated groups likely reflect the increased beta diversity of the gut microbiota as compared to the control group. Indeed, the metabolization of primary BAs into secondary BAs can only be performed in certain bacterial species that have the necessary enzymes, such as 7-alpha-dehydroxylases in bacteria like *C. scindens*, *P. hiranonis*, or *E. hylemonae* [54,56,57]. Therefore, the significantly increased secondary BAs in the feces may reflect the enrichment of these species which may directly compete with *C. difficile*. Previous studies have shown that *C. scindens* is associated with the prognosis of *C. difficile* infection [53,54]. Therefore, the retention of these products in the gut may also contribute to the therapeutic effect of the two compounds. Furthermore, secondary bile acids produced in the gut such as DCA or LCA can act as natural inhibitors for *C. difficile* infection [57]. Taken together, our data suggests that both compounds demonstrated anti-*C. difficile* activity via altering the BA–gut microbiome homeostasis and interaction. However, since this is not a direct observation, future studies should be focused on testing this hypothesis.

## 5. Conclusions

In summary, our data from our previous studies in potency and pharmacokinetics, in conjunction with the results from the present study, indicate that bile acid analogs are promising therapeutics for *C. difficile* infection. Our study provided further evidence that these analogs are safe, and they demonstrate unique interferences with the bile acids EHC and modulate the profiles of key bile acids that critically impact *C. difficile* activity.

## Data Availability

All data produced or examined within this study are comprehensively incorporated in the manuscript.

## Supplementary Materials

The following supporting information can be downloaded at: https://www.mdpi.com/article/10.3390/biom15121672/s1, Figure S1: Ratio of TCA:CDCA among all samples; Figure S2: All bile species among all samples (percent composition); Figure S3: Enrichment analysis of DEGs; Figure S4: Enrichment analysis for upstream regulators; Table S1: Materials/reagents; Table S2: Primer sequences; Table S3: Categorization of bile species; Table S4: Quantities of bile acids present; Table S5: PERMANOVA results for PCA plots; Table S6: DEG results from DESeq2.

## Abbreviations

The following abbreviations are used in this manuscript:

12-keto-LCA	2-keto-lithocholic acid
3-keto,7α,12α(OH)_2_	3-keto-7α,12α-dihydroxy-5β-cholan-24-oic acid
3-keto-LCA	3-keto-lithocholic acid
7-keto-DCA	7-keto-deoxycholic acid
7-keto-LCA	7-keto-lithocholic acid
C4	7α-hydroxy-4-cholesten-3-one
allo-isoLCA	allo-isolithocholic acid
α-MCA	alpha-muricholic acid
β-MCA	beta-muricholic acid
BA	bile acid
CDCA	chenodeoxycholic acid
CDCA-3-S	chenodeoxycholic acid-3-sulfate
CA	cholic acid
CA-3-S	cholic acid-3-sulfate
CA-7-S	cholic acid-7-sulfate
CDI	*Clostridioides difficile* infection
DCA	deoxycholic acid
DCA-3-S	deoxycholic acid-3-sulfate
DMSO	dimethyl sulfoxide
EHC	enterohepatic circulation
Gβ-MCA	glyco-beta-muricholic acid
GCDCA	glycochenodeoxycholic acid
GCA	glycocholic acid
GDCA	glycodeoxycholic acid
GHCA	glycohyocholic acid
GHDCA	glycohyodeoxycholic acid
GLCA	glycolithocholic acid
GUDCA	glycoursodeoxycholic acid
HCA	hyocholic acid
HDCA	hyodeoxycholic acid
isoDCA	isodeoxycholic acid
isoLCA	isolithocholic acid
LC-MS	liquid chromatography- mass spectrometry
LCA	lithocholic acid
LCA-3-S	lithocholic acid-3-sulfate
mSA	metanilic acid
MDCA	murideoxycholic acid
ω-MCA	omega-muricholic acid
PCA	principal component analysis
Tα-MCA	tauro-alpha-muricholic acid
Tβ-MCA	tauro-beta-muricholic acid
Tω-MCA	tauro-omega-muricholic acid
TCDCA	taurochenodeoxycholic acid
TCA	taurocholic acid
TDCA	taurodeoxycholic acid
THDCA	taurohyodeoxycholic acid
TLCA	taurolithocholic acid
TUDCA	tauroursodeoxycholic acid
UDCA	ursodeoxycholic acid
UDCA-3-S	ursodeoxycholic acid-3-sulfate
