# Supplementary material for: Bile Acid Analogs with Anti-Germination Activities for Prophylaxis of Clostridioides difficile Infection Alter Bile Acid Homeostasis in the Enterohepatic Cycle
Source: Biomolecules. 2025 Dec 1;15(12):1672. doi: 10.3390/biom15121672 (PMC12730999; doi:10.3390/biom15121672)
Supplement: Supplementary file 1 [file biomolecules-15-01672-s001.zip › supplementary tables 1 thru 5.pdf]

Supplementary Tables

Table S1

Material/Reagent	Vendor	Catalog Number
RNAprotect Tissue Reagent	Qiagen	76106
RNeasy Mini Kit	Qiagen	74106
TRIzol Reagent	ThermoFisher	15596026
Chloroform	Sigma-Aldrich	C2432
Isopropanol	Sigma-Aldrich	I9516
10% Neutral Buffered Formalin	Epredia	5735
100% Ethanol, Molecular Biology Grade	ThermoFisher	BP28184
Paraffin Wax	Leica	39603002
Hematoxylin	Pureview	16600
Eosin Y	Richard Allen	7111
Xylene	Fisher Scientific	X5SK-4
High-Capacity cDNA Kit	Applied Biosystems	4368813
PowerUp SYBR Green Master Mix	Applied Biosystems	A25742
0.2 mL PCR Tubes (8-tube strips)	Thermo Scientific	AB0620
384-Well PCR Plates (Clear)	Applied Biosystems	4309849
Optical Adhesive Covers	Applied Biosystems	4311971
1.7 mL Rnase-free tubes	Thermo Scientific	3448
UltraPure Water	Invitrogen	10977-015
HistoPrep Ethanol	Fisher Scientific	HC-800-1GAL

Table S2

Primers		
Cyp 27a1	F	CCAGGCACAGGAGAGTACG
	R	GGGCAAGTGCAGCACATAG
Cyp 7a1	F	AGCAACTAAACAACCTGCCAGTACT A
	R	GTCCGGATATTCAAGGATGCA
Cyp 7b1	F	CCGAGAAGTGCAGGATA
	R	TGGAGGAAAGAGGGCTACAA
Bsep	F	TCTGACTCAGTGATTCTTCGCA
	R	CCCATAAACATCAGCCAGTTGT
Fgf15	F	GCTGGTCCCTATGTCTCCAACT
	R	ACCGCGCGGAATTCCAAC
Fxr	F	GCTTGATGTGCTACAAAAGCTG
	R	CGTGGTGATGGTTGAATGTCC
Tgr5	F	TGATGACACCCAACAGCACTGA
	R	GGCTAGTAGTAGGCTTAGGAAGAAG
Asbt	F	GGCTCCAATATCCTGGCCTATTG
	R	GTGGAGCAAGTGGTCATGCTAAC
Ost alpha	F	CCTCACCATCATCTTGACCTTTC
	R	GAGGGCGCGAGGAATC
Ost beta	F	TTGCCTAGCTACAACTGCATTTC
	R	CCATGTTTCTGCTCCTGATTGAGAT
Beta actin	F	GGCTGTATTCCCCTCCATCG
	R	CCAGTTGGTAACAATGCCATGT
Gapdh	F	AGGTCGGTGTGAACGGATTTG
	R	GGGGTCGTTGATGGCAACA

Table S3

Category	Related Species (by column)				
**Primary**	CA	CDCA	UDCA	αMCA	βMCA
**Primary Conjugated**	TCA<br>GCA	TCDCA<br>GCDCA	TUDCA<br>GUDCA	TαMCA	TβMCA<br>GβMCA
**Secondary**	DCA<br>isoDCA<br>7-keto-DCA<br>DCA-3-S<br>CA-3-S<br>CA-7-S	LCA<br>isoLCA<br>Allo-isoLCA<br>3-Keto-LCA<br>7-keto-LCA<br>12-keto-LCA<br>LCA-3-S<br>CDCA-3-S	LCA<br>isoLCA<br>Allo-isoLCA<br>3-Keto-LCA<br>7-keto-LCA<br>12-keto-LCA<br>LCA-3-S<br>UDCA-3-S	MDCA<br>HDCA	MDCA<br>HDCA<br>wMCA<br>3-Keto,7a,12a(OH)2
**Secondary Conjugated**	TDCA<br>GDCA	TLCA<br>GLCA	TLCA<br>GLCA	THDCA<br>GHDCA	THDCA<br>GHDCA<br>GHCA<br>TωMCA

Table S4

Tissue	Treatment	Precursor	Primary	Primary Conjugated	Secondary	Secondary Conjugated
Liver	Control	0	5	7	5	3
	CamSA	0	5	7	6	4
	CA-Quin	0	5	7	7	5
Chyme	Control	1	2	7	2	4
	CamSA	1	2	8	3	5
	CA-Quin	1	3	7	3	4
Feces Day 3	Control	0	1	5	2	3
	CamSA	0	5	5	9	1
	CA-Quin	0	4	5	12	3
Feces Day 7	Control	0	1	5	1	4
	CamSA	0	5	4	7	1
	CA-Quin	0	4	5	9	4

Table S5

PERMANOVA Results for PCA Plots

Liver RNASeq

Comparison	R^2	P value	CI Lower	CI Upper
DMSO vs CamSA	0.16	0.21	0.18	0.51
DMSO vs CA-Quin	0.18	0.17	0.17	0.52

Liver

Comparison	R^2	P value	CI Lower	CI Upper
DMSO vs CamSA	0.3	0.11	0.14	0.80
DMSO vs CA-Quin	0.26	0.16	0.18	0.73

Ileum

Comparison	R^2	P value	CI Lower	CI Upper
DMSO vs CamSA	0.16	0.18	0.11	0.60
DMSO vs CA-Quin	0.20	0.02	0.14	0.70

Feces Day 3

Comparison	R^2	P value	CI Lower	CI Upper
DMSO vs CamSA	0.46	0.02	0.30	0.80
DMSO vs CA-Quin	0.49	0.03	0.37	0.75

Feces Day 7

Comparison	R^2	P value	CI Lower	CI Upper
DMSO vs CamSA	0.78	0.03	0.62	0.93
DMSO vs CA-Quin	0.57	0.03	0.33	0.86
