# Supplementary figures and images for: Bile Acid Analogs with Anti-Germination Activities for Prophylaxis of Clostridioides difficile Infection Alter Bile Acid Homeostasis in the Enterohepatic Cycle
Source: Biomolecules. 2025 Dec 1;15(12):1672. doi: 10.3390/biom15121672 (PMC12730999; doi:10.3390/biom15121672)

Figure S1

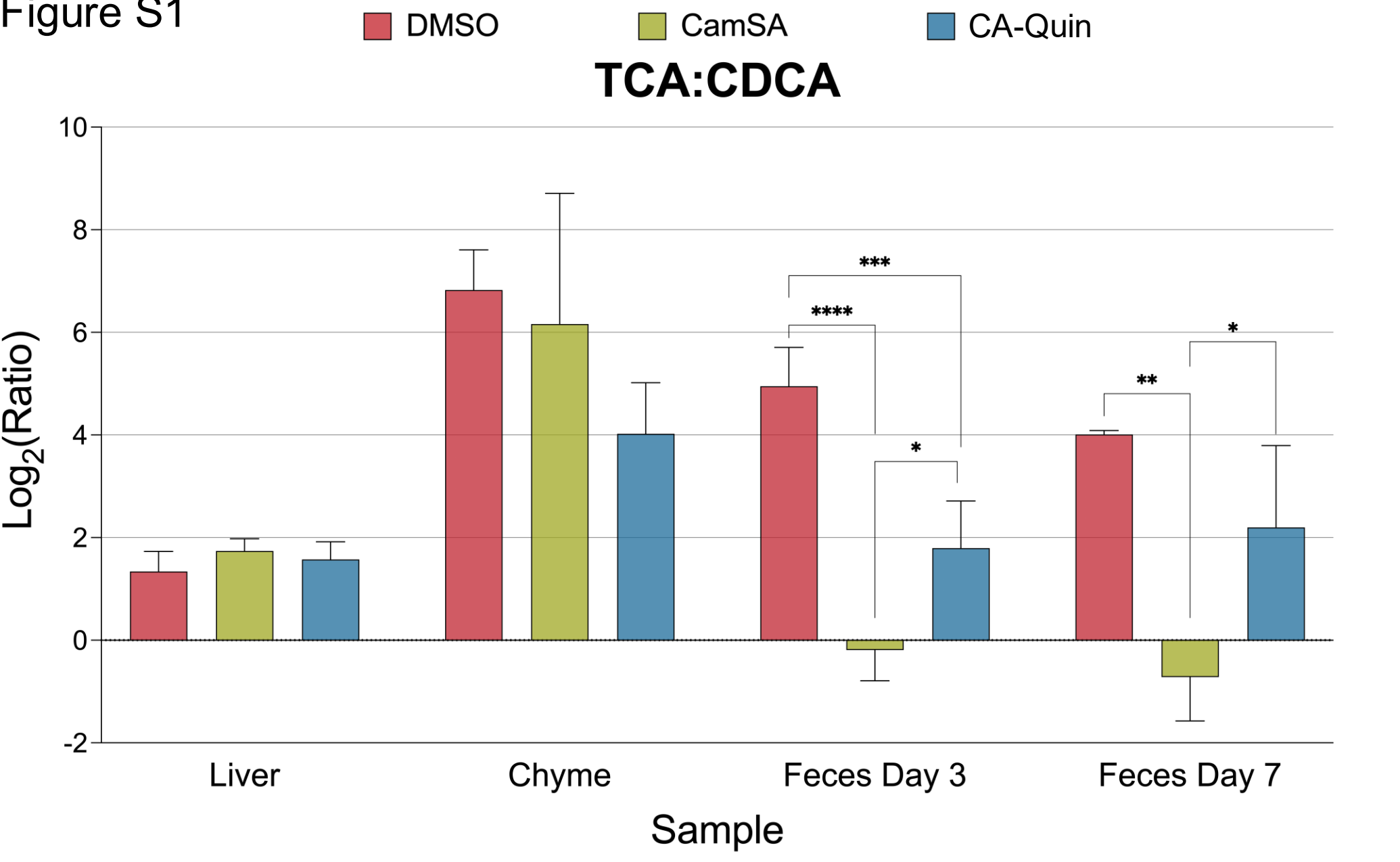

Supplement: Supplementary file 1 [file biomolecules-15-01672-s001.zip › supplementary figure 1.pdf]

Figure S2 All Bile Species Among All Samples

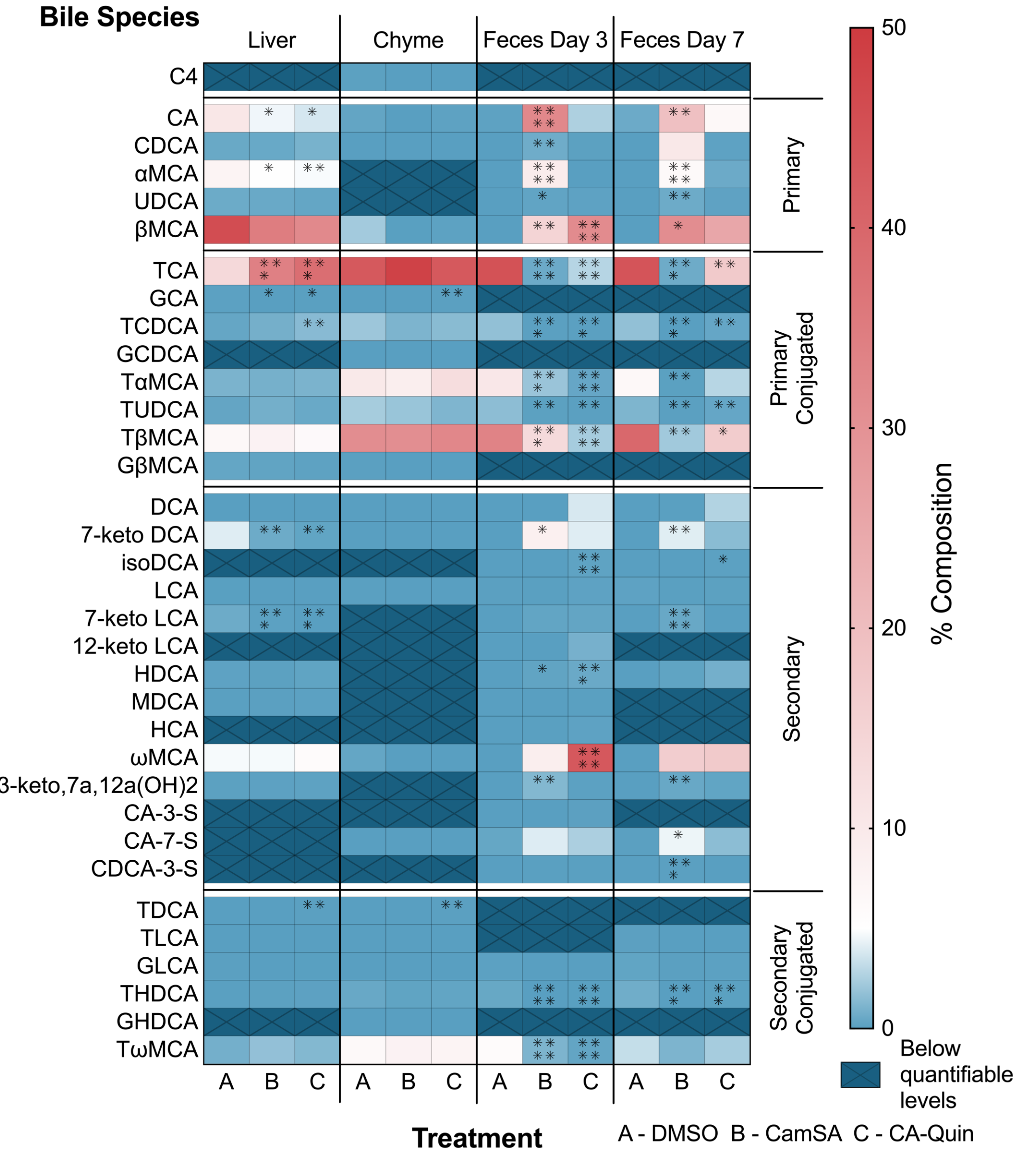

Supplement: Supplementary file 1 [file biomolecules-15-01672-s001.zip › supplementary figure 2.pdf]

Figure S3

A CamSA

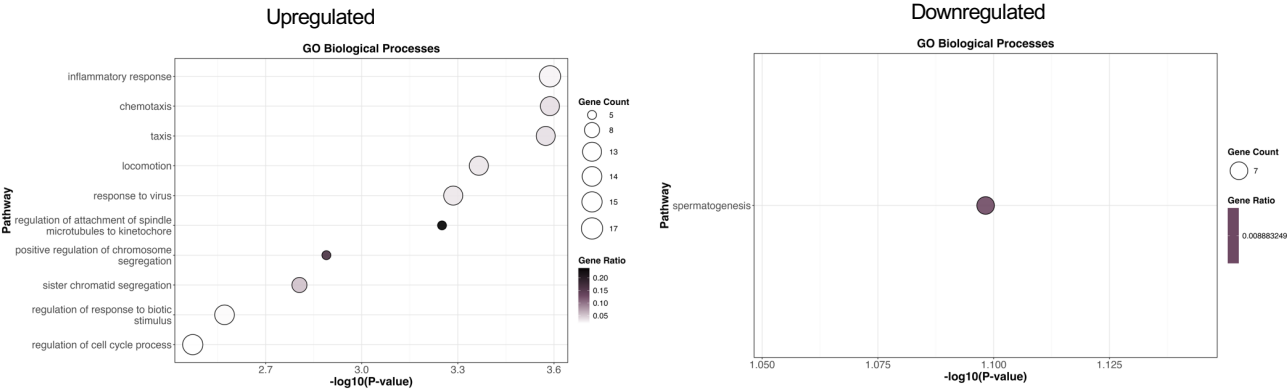

B CA-Quin

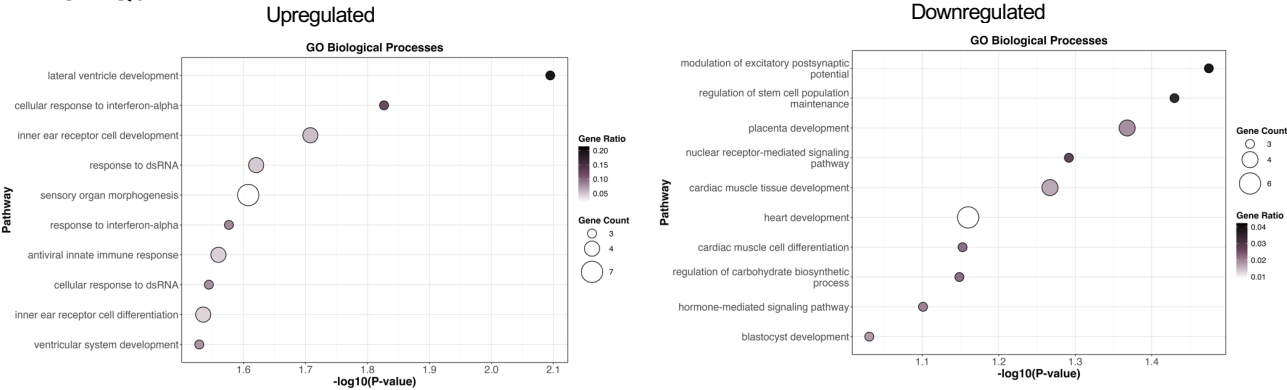

C Shared

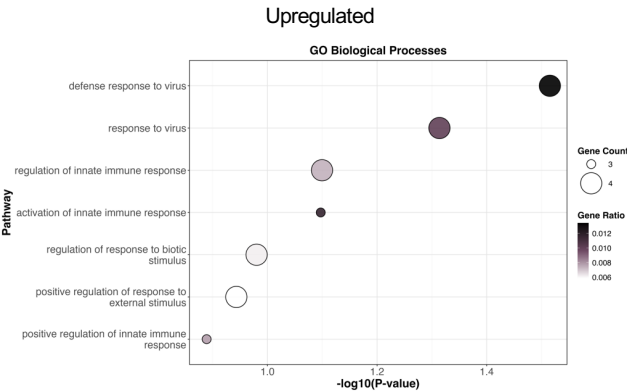

Supplement: Supplementary file 1 [file biomolecules-15-01672-s001.zip › supplementary figure 3.pdf]

Figure S4

A

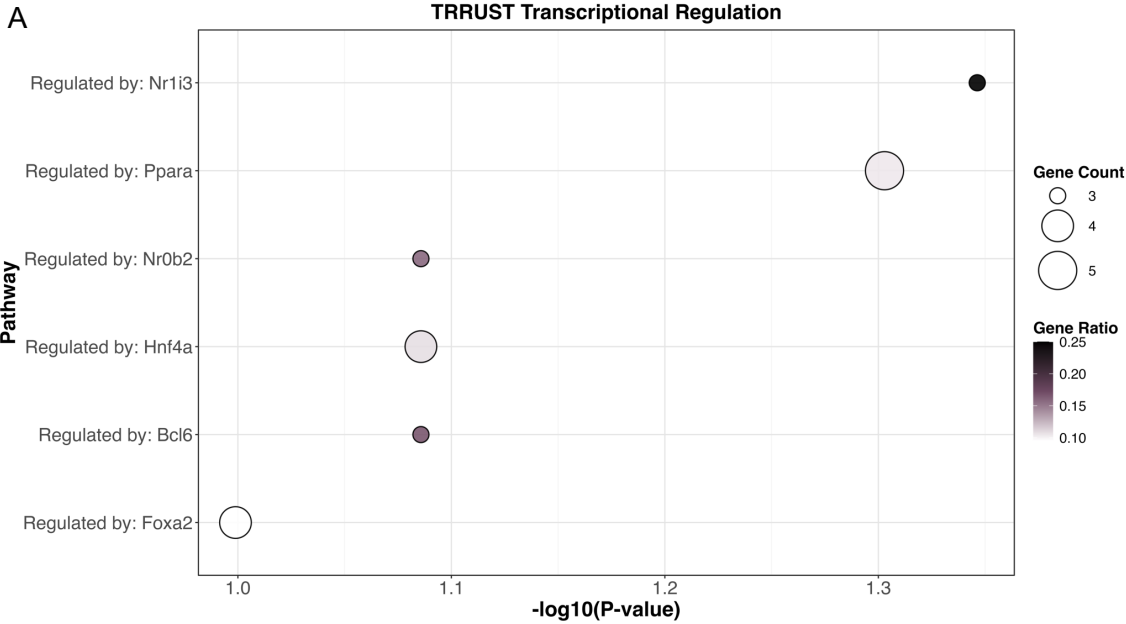

B

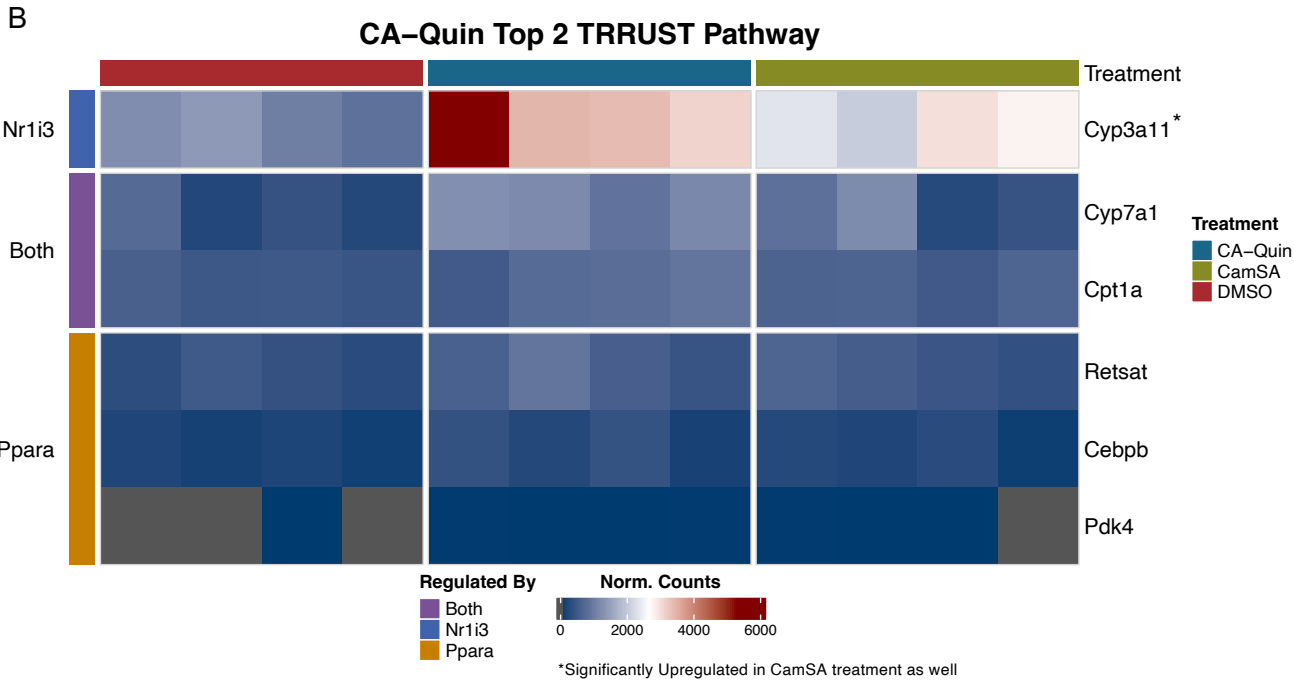

Supplement: Supplementary file 1 [file biomolecules-15-01672-s001.zip › supplementary figure 4.pdf]
